# A Rare Coexistence: Uterine Leiomyoma Arising in a Mature Cystic Teratoma and a Pedunculated Endometriotic Cyst Mimicking Leiomyoma—Two Case Reports

**DOI:** 10.1002/ccr3.73001

**Published:** 2026-06-21

**Authors:** Mena Abdalla, Victoria Barker, Anitha Nayar, Sahathevan Sathiyathasan, Adjoa Appiah, Ibrahim ElHag, Alexander Steshenko, Saira Khan, Arjun Anilkumar, Aisha Hameed

**Affiliations:** ^1^ Princess Royal University Hospital, King's College Hospital NHS Foundation Trust London UK; ^2^ Queen's University Belfast Belfast Northern Ireland UK

**Keywords:** case reports, endometriosis, leiomyoma, teratoma, uterine neoplasms

## Abstract

Uterine leiomyomas are common benign tumors, but their clinical presentation can be misleading. We report two unusual cases of pedunculated uterine masses presenting with heavy vaginal bleeding. In the first case, a woman in her 40s underwent urgent resection of a prolapsing mass after acute hemorrhage; histopathological examination demonstrated a mature cystic teratoma containing a leiomyomatous component, an exceptionally rare finding. In the second case, a woman in her late 40s presented with a pedunculated cervical mass initially considered to be a leiomyoma; histopathology instead showed an endometriotic cyst with no definitive evidence of leiomyoma. These cases emphasize that apparently typical prolapsing uterine masses may conceal rare pathological entities and highlight the importance of comprehensive histopathological assessment for accurate diagnosis, counseling, and management.

## Key Clinical Message

These two cases illustrate how prolapsing uterine or cervical masses with heavy vaginal bleeding can conceal unexpected pathology. One case represents, to our knowledge, the first reported uterine leiomyoma arising within a mature cystic teratoma, while the second demonstrates an endometriotic cyst masquerading clinically as a pedunculated leiomyoma. Together, they reinforce that definitive diagnosis depends on careful histopathological examination.

## 1 Background

Uterine leiomyomas are the most common tumors in women worldwide and affect more than 70% of women over their lifetime [1]. When submucosal and pedunculated, they may prolapse through the cervix and present with heavy vaginal bleeding, pain, or a visible vaginal mass, often prompting urgent surgical management [2].

Primary uterine teratomas are exceptionally rare, with only a small number of uterine corpus cases reported in the literature [3]. Although transformation within mature cystic teratomas is recognized, secondary neoplasms are uncommon and are more often malignant than benign [4]. Endometriosis may also present as a mass‐forming lesion and, in its polypoid forms, can mimic other gynecological tumors or even malignancy [5].

Against this background, the two cases presented here demonstrate how seemingly typical prolapsing uterine masses may conceal unexpected pathology and why histopathological confirmation remains essential.

## 2 Case 1: Leiomyoma Arising in Mature Cystic Teratoma

### 2.1 Case Presentation

A woman in her 40s (para 2) presented to the emergency department with a one‐day history of heavy vaginal bleeding on the third day of her menstrual cycle. She reported longstanding menorrhagia but no other gynecological complaints. Her obstetric history included two spontaneous vaginal deliveries, and her last cervical smear was performed over 20 years ago. She had no significant medical history and was not on regular medication.

On examination, she appeared pale and distressed. A 2–3 cm irregular, prolapsed mass was visible at the external cervical os with active bleeding. Approximately 200 mL of blood loss was observed during examination. Bimanual assessment confirmed a polypoid mass protruding through the cervix with a palpable stalk. Laboratory investigations revealed a hemoglobin drop from 8.4 to 6.0 g/dL within 24 h, consistent with acute blood loss anemia.

Emergency management included insertion of a vaginal pack and urinary catheter. She was scheduled for urgent examination under anesthesia (EUA) with planned resection of the prolapsed mass, with consent obtained for possible hysterectomy if bleeding was uncontrolled.

### 2.2 Investigations

- Laboratory: Severe microcytic anemia consistent with iron deficiency secondary to chronic menorrhagia. Coagulation profile normal. Serum β hCG negative.
- Imaging (post‐operative TVUS): Anteverted uterus of normal size (8.5 × 5.2 × 4.8 cm), heterogeneous myometrium suggestive of adenomyosis, and a small posterior intramural fibroid (17 × 13 × 7 mm). Endometrium thin (5 mm), appropriate for cycle phase. Ovaries normal bilaterally; no adnexal masses or free fluid. Cervical canal normal post procedure.
- Preoperative assessment: Limited due to emergency presentation; immediate surgical intervention prioritized.

### 2.3 Differential Diagnosis

Primary Considerations: The clinical presentation of a prolapsed pedunculated mass with heavy bleeding in a woman in her 40s initially suggested several differential diagnoses:

1. Pedunculated submucosal leiomyoma—most likely preoperative diagnosis.
2. Endometrial polyp—considered but less consistent with mass size/consistency.
3. Cervical polyp—excluded due to uterine origin.
4. Malignant uterine neoplasm—considered given age and irregular mass appearance; histology required.
5. Retained products of conception—excluded by negative pregnancy test.

Post histology: Unexpected diagnosis of mature cystic teratoma with leiomyomatous elements, underscoring the importance of comprehensive histopathological evaluation.

### 2.4 Treatment

EUA revealed a 6 × 3 cm pedunculated mass arising from the uterine fundus. Resection was performed at the stalk using diathermy, with thorough cauterization for hemostasis. Estimated intraoperative blood loss was < 50 mL. A small vaginal pack was inserted prophylactically and removed prior to discharge. She was prescribed tranexamic acid and norethisterone for residual bleeding control and discharged the same day.

### 2.5 Outcome and Follow‐Up

#### 2.5.1 Outcome

The patient had an uneventful postoperative recovery and was discharged home on the day of surgery. At her follow‐up appointment, she reported complete resolution of her heavy bleeding and had returned to her normal activities. She was counseled about the histological findings and the need for continued gynecological surveillance. Long‐term follow‐up will include monitoring of the small residual intramural fibroid and routine cervical screening.

#### 2.5.2 Histopathological Findings

The resected specimen underwent comprehensive histopathological examination. The final report revealed a complex nodule partially covered by unremarkable squamous epithelium with areas of focal ulceration and associated acute inflammation. Within the nodule, multiple cystic spaces lined by keratinizing squamous epithelium were identified, with adjacent skin appendage structures clearly visible.

The specimen demonstrated remarkable tissue diversity, containing adipose tissue, sheets of glial tissue, ganglion cells, cartilage, bone, and glandular epithelium resembling both large bowel and respiratory epithelium—features characteristic of a mature cystic teratoma containing elements from all three germ layers (ectoderm, mesoderm, and endoderm).

**FIGURE 1:**
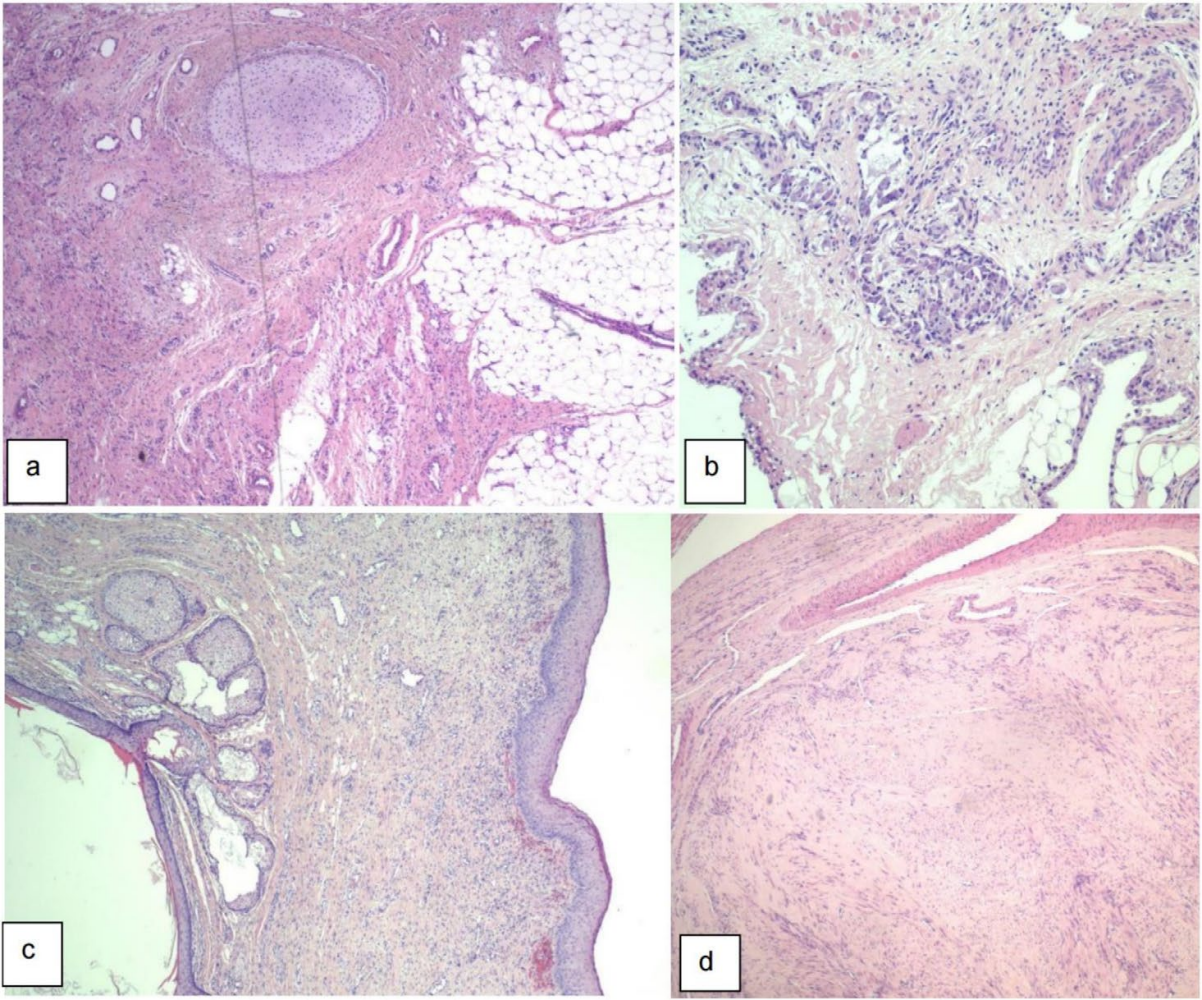
Photomicrographs of lesion, case 1. (a) Mature cartilage and adipose tissue, (b) Ganglion cells and adipose tissue, (c) Squamous epithelium and sebaceous glands, (d) Leiomyomatous component.

Significantly, there was marked proliferation of smooth muscle fibers with focal hyalinization, which stained positively with desmin immunohistochemistry, confirming the smooth muscle origin. These features were consistent with a diagnosis of leiomyoma arising within a mature cystic teratoma. Importantly, no immature elements were identified, and there were no signs of malignancy in any component of the specimen (Figure 1).

## 3 Case 2: Pedunculated Mass with Endometriotic Degeneration

### 3.1 Case Presentation

A woman in her late 40s presented to the emergency department with heavy vaginal bleeding and clots, ongoing for approximately 18 months and worsening in recent months. She had recently commenced tranexamic acid and norethisterone prescribed by her general practitioner, with minimal relief.

Her gynecological history included increasingly irregular periods over the past two years, consistent with perimenopausal transition. A Mirena intrauterine system had been inserted several months earlier without improvement in symptoms. Obstetric history included spontaneous vaginal deliveries, though parity was unclear.

Past medical history included anxiety, eczema, folic acid deficiency, and migraines. She was allergic to penicillin and a social smoker. Notably, she had undergone hysteroscopy with endometrial polypectomy and laparoscopy several months earlier.

On speculum examination, Mirena threads were not visible. A smooth 5–6 cm mass was observed obstructing the cervix. Vaginal examination confirmed a mass of similar dimensions protruding through the cervix. She was scheduled for urgent transvaginal ultrasound (TVUS) and hysteroscopy.

### 3.2 Investigations

- Laboratory: Mild anemia consistent with chronic blood loss; iron studies confirmed iron deficiency. Inflammatory markers were normal.
- Microbiology: Genital swabs (high vaginal, endocervical, chlamydia/gonorrhea NAAT) were negative. Previous STI screening was normal.
- Imaging: Patient reported TVUS showed a normally positioned Mirena coil and no uterine abnormalities; formal report unavailable.
- Hormonal assessment: Elevated FSH and LH with fluctuating estradiol levels, consistent with perimenopausal transition.

### 3.3 Differential Diagnosis

Primary considerations included:

1. Pedunculated submucosal leiomyoma—leading preoperative diagnosis based on clinical presentation and mass characteristics.
2. Endometrial polyp—recurrence possible given prior history, though mass size/consistency suggested otherwise.
3. Adenomyomatous polyp—benign lesion with similar presentation.
4. Endometrial hyperplasia/carcinoma—considered due to age, chronic bleeding, and failed hormonal therapy.
5. Cervical pathology—less likely given apparent uterine origin.
6. Endometriotic lesion—not initially suspected but a possible atypical presentation.
7. Inflammatory conditions—considered unlikely.

Post histology: Diagnosis of endometriotic cyst was unexpected, underscoring the importance of tissue sampling and histopathological confirmation in abnormal uterine bleeding.

### 3.4 Treatment

Hysteroscopy and polypectomy under general anesthesia revealed a 5 × 5 cm pedunculated mass with a narrow stalk protruding through the cervix. The mass was resected, and the distorted Mirena coil was removed. Estimated blood loss was < 50 mL. Postoperatively, contraceptive counseling and follow up were arranged.

#### 3.4.1 Outcome and Follow Up

Five days post procedure, the patient presented with abdominal pain, dysuria, and persistent bleeding. Clinical assessment excluded endometritis; she was discharged with reassurance, safety netting advice, and instructions to restart hormonal therapy if bleeding persisted beyond six weeks.

#### 3.4.2 Histopathology

- Endometrial specimen: inactive glands, pseudodecidualized stroma, scattered acute inflammatory cells; no hyperplasia, atypia, or malignancy.
- Resected mass: cyst wall lined by cuboidal epithelium, hemosiderin laden macrophages, occasional glands; consistent with endometriotic cyst. No features of leiomyoma or malignancy identified.

### 3.5 Outcome and Follow‐Up

#### 3.5.1 Outcome

The patient presented to the emergency department five days post‐hysteroscopy with abdominal pain, dysuria, and persistent vaginal bleeding, raising concern for possible endometritis. However, after clinical assessment, she was reassured and discharged home without antibiotics, with safety‐netting advice to restart hormonal treatment if bleeding persisted beyond six weeks and to seek gynecological review if symptoms continued.

#### 3.5.2 Histopathological Findings

**FIGURE 2:**
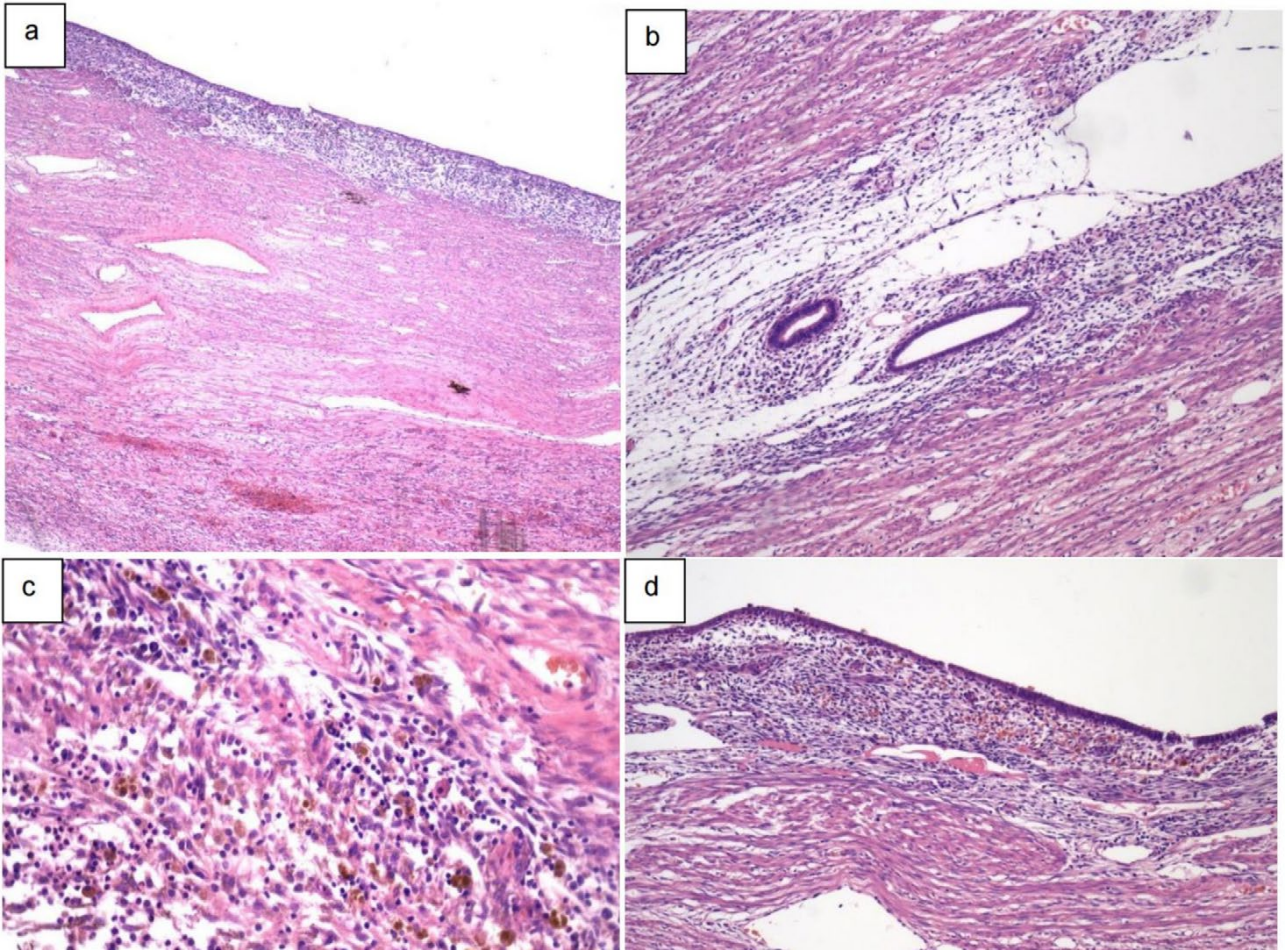
Photomicrographs of the lesion, case 2. (a) Section of cyst wall showing epithelium with underlying fibrous stroma, (b) Endometrioid glands within the cyst wall, (c) Haemosiderin laden macrophages, (d) Higher power view showing cuboidal lining epithelium.

Histopathological examination of the endometrial specimen showed features suggestive of exogenous progestational effect, with inactive endometrial glands, pseudodecidualization of the stroma, and scattered acute inflammatory cells. No evidence of hyperplasia, atypia, or malignancy was identified (Figure 2).

The resected mass showed sections of cyst wall lined by a single layer of cuboidal epithelium with scattered inflammatory cells. Hemosiderin‐laden macrophages were present throughout the specimen, and occasional glands were noted within the cyst wall. These features were consistent with an endometriotic cyst rather than a typical leiomyoma. Notably, no definitive features of leiomyoma were identified in the specimen, and there was no evidence of malignancy.

## 4 Discussion

These two cases illustrate the diagnostic complexity that can arise in seemingly straightforward presentations of pedunculated uterine masses. Both patients presented with heavy vaginal bleeding and prolapsed cervical lesions, yet histopathological analysis revealed entirely different and unexpected pathological entities.

### 4.1 Case 1: Leiomyoma Within a Mature Cystic Teratoma

The identification of a leiomyoma arising within a mature cystic teratoma of the uterus represents an unprecedented finding. Somatic transformation of mature cystic teratomas is recognized, most commonly involving malignant change—particularly squamous cell carcinoma in approximately 1%–2% of cases [6]. Benign secondary neoplasms within teratomas are exceptionally rare, with only a handful of reports, predominantly in ovarian teratomas [7].

The pathogenesis of this rare combination remains uncertain. The leiomyoma may have arisen from smooth muscle elements within the teratoma, representing somatic overgrowth rather than true neoplastic transformation. Alternatively, this could represent a collision tumor, with two distinct pathological processes occurring concurrently. Positive desmin staining confirmed the smooth muscle origin of the leiomyomatous component, while diverse tissue elements established the teratomatous nature of the lesion.

This case underscores three key points: (1) common clinical presentations can conceal rare pathological entities; (2) comprehensive histopathological examination of all resected specimens is essential; and (3) benign secondary neoplasms can expand our understanding of uterine teratoma biology.

### 4.2 Case 2: Endometriotic Cyst Mimicking Leiomyoma

The second case demonstrates how endometriotic cysts can clinically and macroscopically mimic pedunculated leiomyomas. Histopathology revealed hemosiderin laden macrophages and characteristic cyst wall architecture, confirming endometriotic origin. This highlights the importance of considering endometriosis in the differential diagnosis of pedunculated uterine masses, particularly in perimenopausal women with chronic pelvic symptoms.

The patient's prior gynecological procedures and Mirena coil may have contributed to lesion development, though the exact pathogenesis remains unclear. This case emphasizes the potential for diagnostic confusion when relying solely on clinical and imaging findings, reinforcing the necessity of histopathological confirmation.

### 4.3 Clinical Implications

Together, these cases highlight several important principles:

- Even routine gynecological presentations may conceal rare or unexpected pathology.
- Preoperative imaging has limitations in characterizing complex uterine lesions.
- Histopathological examination remains the gold standard for definitive diagnosis.
- Conservative surgical approaches can effectively manage complex pathology while preserving fertility and uterine function.

These cases expand the spectrum of uterine pathology and reinforce the importance of vigilance, thorough investigation, and reliance on histology in gynecological practice.

## 5 Learning Points/Take Home Messages

- Pedunculated uterine masses can harbor unexpected and extremely rare pathological findings, including the first reported case of leiomyoma arising within a uterine mature cystic teratoma.
- Endometriotic cysts can clinically mimic pedunculated leiomyomas, emphasizing the importance of histopathological examination for accurate diagnosis.
- Comprehensive histopathological evaluation is essential for all resected uterine specimens, as complex pathology may not be apparent from clinical presentation or imaging findings.
- Conservative surgical management can be effective for complex uterine pathology while preserving reproductive function.
- Clinicians should maintain awareness of rare pathological entities and the potential for diagnostic surprises in common clinical presentations.

## Author Contributions

**Mena Abdalla:** conceptualization, formal analysis, investigation, methodology, supervision, writing – original draft. **Victoria Barker:** conceptualization, investigation, visualization, writing – original draft. **Anitha Nayar:** investigation, visualization, writing – original draft. **Sahathevan Sathiyathasan:** methodology, supervision, writing – original draft. **Adjoa Appiah:** data curation, supervision, validation, writing – original draft. **Ibrahim ElHag:** resources, visualization, writing – original draft. **Alexander Steshenko:** investigation, methodology, writing – review and editing. **Saira Khan:** investigation, methodology, writing – original draft. **Arjun Anilkumar:** data curation, investigation, visualization. **Aisha Hameed:** conceptualization, data curation, formal analysis, methodology, supervision.

## Funding

The authors have nothing to report.

## Consent

Written informed consent was obtained from the patients for publication of this case report and any accompanying images. A copy of the written consent is available for review by the Editor‐in‐Chief of this journal.

## Conflicts of Interest

The authors declare no conflicts of interest.

## Data Availability

All data is available upon request from the corresponding author.
